# Cervical cancer with bilateral ovarian metastases: case report and review of literature

**DOI:** 10.1259/bjrcr.20180047

**Published:** 2018-06-01

**Authors:** Menge Kuria, Samuel Gitau, Khadija Warfa

**Affiliations:** 1 Department of Radiology and Imaging, Aga Khan University Hospital, Nairobi, Kenya; 2 Department of Obstretics and Gynaecology, Aga Khan University Hospital, Nairobi, Kenya

## Abstract

Cervical cancers only rarely metastasize to the ovaries. Most of the reported cases are in Western and Asian literature and to the best of our knowledge, this is the first case report of cervical cancer presenting with bilateral ovarian metastasis in sub-Saharan Africa. We present a case report of a 44-year-old female with an 8-month history of irregular per vaginal bleeding. Imaging showed ill-defined masses in the cervix and both adnexae. After biopsy, a diagnosis of cervical squamous cell carcinoma with bilateral ovarian metastases was made.

## Introduction

Cervical cancers rarely metastasize to the ovaries. The proportion of cases presenting with ovarian metastases at the time of surgery ranges from 0.6 to 1.5%.^1^ In terms of histological type, adenocarcinomas are more likely to metastasize to the ovaries than squamous cell carcinomas (SCCs). In different case series, 5–8% of cervical adenocarcinomas *v* *s* 0.4–1.3% of SCCs metastasized to ovary.^1–3^ We present a rare case of cervical SCC with bilateral ovarian metastasis in a 44-year-old female. The fact that the histological type is SCC and affects both ovaries makes this case even more unique.

## Case report

CW is a 44-year-old female with four living children, who presented with an 8-month history of abnormal vaginal bleeding that started as intramenstrual spotting progressed to copious post-coital bleeding at presentation. She had occasional abdominal cramps and early satiety and no history of weight changes or constipation. No other systemic manifestations were elicited; she had a PAP smear test 6 years earlier that was normal. On clinical exam, the only positives were palpable abdominopelvic mass measuring 8 cm and a cervical tumour 3 cm with no evidence of parametrial, pelvic side wall or rectal involvement.

**Figure 1.**
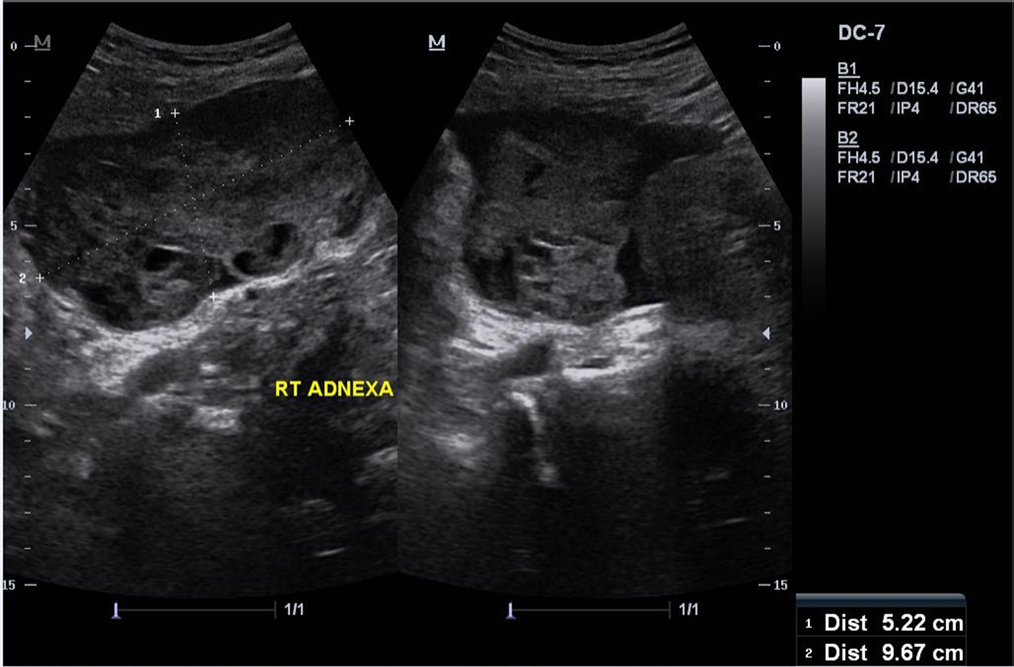
Pelvic ultrasound images demonstrate a heterogeneous cystic mass with solid components measuring 5 × 9 cm in the right adnexa. There is associated ascites noted.

An ultrasound revealed a complex cystic mass with vascular mural nodules and ascites seen within the right adnexa (Figure 1).

**Figure 2.**
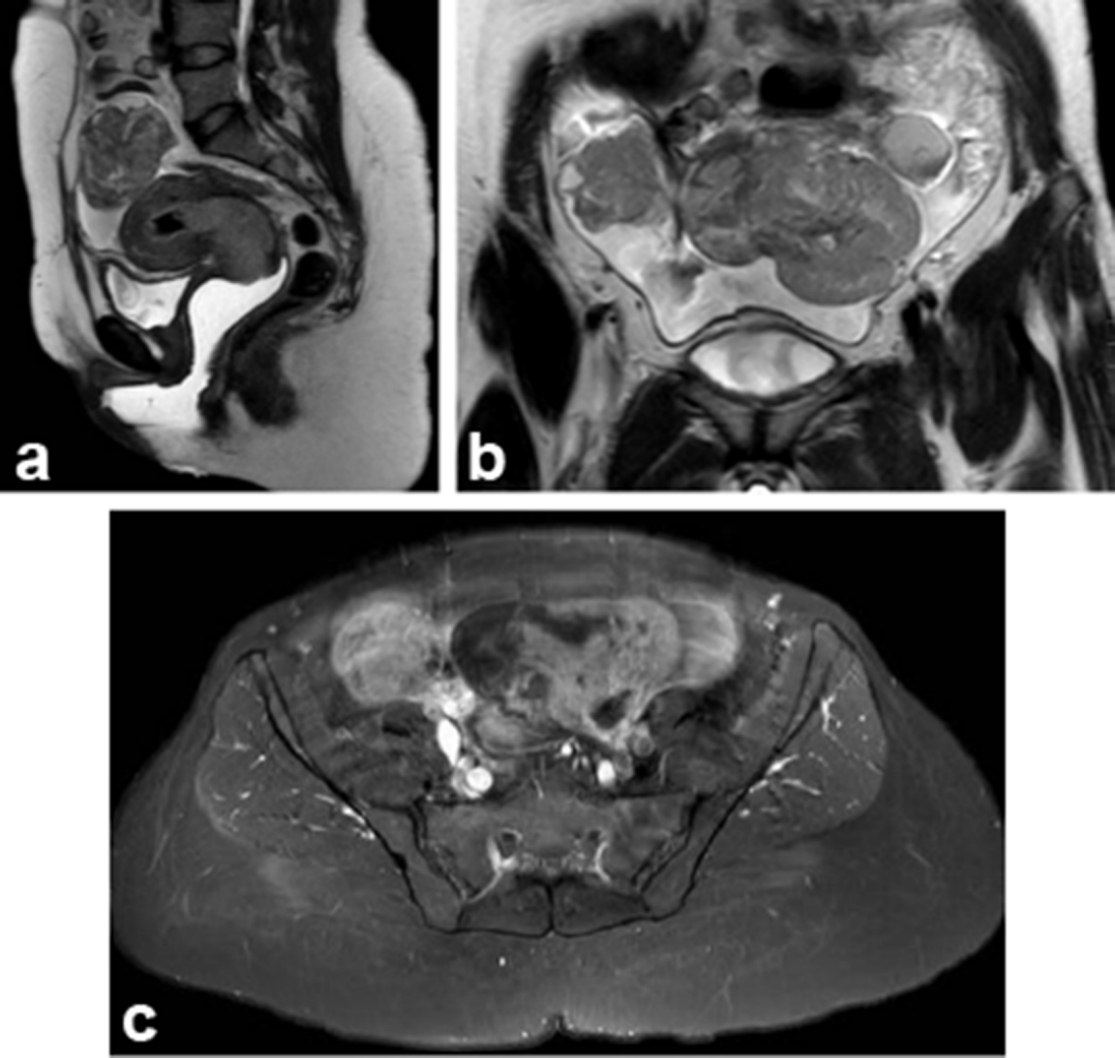
(a) A sagittal *T*_2_W image (a) demonstrates hyperintense cervical mass with endoluminal extension into the endometrial cavity. The mass is causing obliteration of anterior fornix of the vagina. The lower vagina is normal. There is no evidence of bladder or rectal invasion. (b) Coronal *T*_2_W demonstrates complex masses arising from both adnexae with ascites. (c) Post-contrast fat sat *T*_1_W axial image shows bilateral adnexal complex lesions, with enhancement of the solid components. *T*_1_W, *T*_1_ weighted; *T*_2_W, *T*_2_ weighted.

A bulky uterine cervix with a thickened endometrium that was heterogeneous in appearance was also noted. A staging MRI pelvis was also done (Figure 2). A heterogeneous mass was seen arising from the cervix with endoluminal extension into the endometrial cavity. The mass was causing obliteration of the anterior fornix of the vagina with evidence of parametrial invasion anteriorly and on the left. The lower third of the vagina was normal and there was no evidence of bladder or rectal invasion. Also seen were complex masses with cystic and solid components arising from both ovaries. Moderate amount of ascites, peritoneal deposits and para-aortic lymphadenopathy were also seen. The rest of the solid and hollow abdominopelvic viscera including the visualized bone marrow signals were unremarkable. Provisional diagnosis at this stage was cervical malignancy with evidence of parametrial invasion (FIGO Stage IIb) and bilateral complex adnexal masses with ascites and peritoneal deposits suggestive of primary ovarian malignancy.

Biopsy of the cervix was SCC of the cervix and an ultrasound-guided biopsy of the ovarian mass was a SCC. The patient was stages as metastatic cervical cancer. Following tumour board discussion, the patient was started to systemic therapy with carboplatin and paclitaxel to reduce the disease burden.

## Discussion

Cervical cancer is one of the rare cause of metastasis to the ovary with a literature review of published studies indicating that the incidence of ovarian metastasis from uterine cervical cancer ranges between 0.6 and 1.5%.^1^ Varying proportions are noted when the histopathology type is considered. Adenocarcinomas are more likely to metastasize to the ovaries than SCCs. In different case series, 5–8% of cervical adenocarcinomas *v* *s* 0.4–1.3% of SCCs metastasized to ovary.^1–3^ Our patient had histology of SCC.

Most of the reported metastases to the ovary are usually microscopic, unilateral, confined to ovarian parenchyma and detected post-operatively.^4, 5^

The mean age of presentation with ovarian metastases is 57.4 years for SCC and 50.2 years for adenocarcinoma,^6^ mostly presenting with signs and symptoms related to the cervical lesion (vaginal bleeding, pelvic pain or abnormal cytology).^2^ Our patient presented earlier at 45 years with an 8-month history of irregular per vaginal bleeding.

There are three ways of carcinomatous spread from the cervix to the ovaries namely haematogenous, lymphatic or transtubal implantation.^7^ Lymphatic spread is undoubtedly the most common pathway with the ovaries having communicating channels with an extensive network of lymphatic channels and nodes in the pelvis. This means that if any pelvic node is involved, retrograde flow or collateral circulation may occur resulting in involvement of the ovaries. Our patient had disease spread to pelvic and para-aortic lymph nodes which implies that the spread to the ovaries was likely through lymphatics.

A study by Webster DR and Sabbadini E^8^ demonstrated that cancer cells were present in the blood of most cancer patients. For the cervical carcinoma in particular, the most common hematogenously metastasized organs are lung, liver, and bone. Because our patient did not have distant organ metastasis, we can infer that haematogenous spread is less favourable in our case.

Due to the strong association between the uterine corpus involvement and ovarian metastasis, transtubal implantation has also been postulated as a mechanism of spread.^9^ In a study by Kim et al^10^ aimed at investigating various risk factors for ovarian metastasis in cervical cancer including age, histologic types, stromal invasion, FIGO stages, lymph node metastasis, parametrial invasion, involvement of the upper vagina and uterine corpus, it was concluded that uterine corpus involvement in addition to histologic type were the only independent risk factors for ovarian metastasis. Our patient had endoluminal extension into the endometrial cavity and thus, transtubal implantation might have also been a plausible route of spread.

## Learning points

1. Prior to diagnosing a primary SCC of the ovary, consideration of possible spread from a cervical tumour should be made.
2. Majority of SCCs of the ovary develop in the background of pre-existing conditions including endometriotic or dermoid cysts. It is, therefore, crucial to try and identify such components that may help in determining the primary nature of the neoplasm.
3. When both ovaries are involved by SCC, evidence strongly dictates this being of metastatic origin although there is a rare possibility of ovarian SCC and SCC *in situ* of the cervix being independent primary neoplasms and should also be considered.
